# Trends and Predictors of COVID-19 Information Sources and Their Relationship With Knowledge and Beliefs Related to the Pandemic: Nationwide Cross-Sectional Study

**DOI:** 10.2196/21071

**Published:** 2020-10-08

**Authors:** Shahmir H Ali, Joshua Foreman, Yesim Tozan, Ariadna Capasso, Abbey M Jones, Ralph J DiClemente

**Affiliations:** 1 Department of Social & Behavioral Sciences School of Global Public Health New York University New York, NY United States; 2 Ophthalmology, Department of Surgery University of Melbourne Melbourne Australia; 3 Global Health Program School of Global Public Health New York University New York, NY United States; 4 Department of Epidemiology School of Global Public Health New York University New York, NY United States

**Keywords:** COVID-19, coronavirus, pandemic, outbreak, infectious disease, social media, information seeking behaviors, surveillance

## Abstract

**Background:**

During the COVID-19 pandemic, there is a heightened need to understand health information seeking behaviors to address disparities in knowledge and beliefs about the crisis.

**Objective:**

This study assessed sociodemographic predictors of the use and trust of different COVID-19 information sources, as well as the association between information sources and knowledge and beliefs about the pandemic.

**Methods:**

An online survey was conducted among US adults in two rounds during March and April 2020 using advertisement-based recruitment on social media. Participants were asked about their use of 11 different COVID-19 information sources as well as their most trusted source of information. The selection of COVID-related knowledge and belief questions was based on past empirical literature and salient concerns at the time of survey implementation.

**Results:**

The sample consisted of 11,242 participants. When combined, traditional media sources (television, radio, podcasts, or newspapers) were the largest sources of COVID-19 information (91.2%). Among those using mainstream media sources for COVID-19 information (n=7811, 69.5%), popular outlets included CNN (24.0%), Fox News (19.3%), and other local or national networks (35.2%). The largest individual information source was government websites (87.6%). They were also the most trusted source of information (43.3%), although the odds of trusting government websites were lower among males (adjusted odds ratio [AOR] 0.58, 95% CI 0.53-0.63) and those aged 40-59 years and ≥60 years compared to those aged 18-39 years (AOR 0.83, 95% CI 0.74-0.92; AOR 0.62, 95% CI 0.54-0.71). Participants used an average of 6.1 sources (SD 2.3). Participants who were male, aged 40-59 years or ≥60 years; not working, unemployed, or retired; or Republican were likely to use fewer sources while those with children and higher educational attainment were likely to use more sources. Participants surveyed in April were markedly less likely to use (AOR 0.41, 95% CI 0.35-0.46) and trust (AOR 0.51, 95% CI 0.47-0.56) government sources. The association between information source and COVID-19 knowledge was mixed, while many COVID-19 beliefs were significantly predicted by information source; similar trends were observed with reliance on different types of mainstream media outlets.

**Conclusions:**

COVID-19 information source was significantly determined by participant sociodemographic characteristics and was also associated with both knowledge and beliefs about the pandemic. Study findings can help inform COVID-19 health communication campaigns and highlight the impact of using a variety of different and trusted information sources.

## Introduction

As of May 2020, the United States has experienced the most severe COVID-19 outbreak of any country in terms of confirmed numbers of cases and deaths [1]. The economic and social disruptions imposed by measures to contain the disease have been significant, with marked spikes in unemployment, poverty, and psychological suffering [2,3]. As such, authorities face the difficult task of convincing the public that compliance with these measures are justified and necessary to circumvent a potentially worse crisis. Therefore, the manner in which information is formulated, the channels through which it is disseminated, and the populations that are targeted must be considered when developing messaging and designing and implementing risk communication strategies.

In the current information age, there is an ever-growing multiplicity of available sources for health-related information, and the public has tended to shift in recent years from reliance on primarily mainstream news outlets toward other sources of information, including social media [4,5]. Sources of information vary in terms of their reliability, completeness, and verifiability, and in the context of the highly polarized political climate in the United States during an election year, antiscientific rhetoric and political bias may underpin reporting by many information outlets [6]. Of particular concern are social media and other online platforms that are not subject to peer review, fact-checking, or compliance with industry regulations to which mainstream sources are usually held [7]. For instance, an analysis of content on Twitter reported that one-quarter of tweets about COVID-19 contained misinformation [7]. During past infectious disease outbreaks, mainstream media sources such as television or newspaper outlets have been significant sources of information [8-11]. However, findings on the most trusted sources of information during such outbreaks have been mixed and have included health officials [8], television [8], the internet [12], and government [12].

During the rapid escalation of the COVID-19 pandemic in March and April 2020, we conducted an online survey on the sources of information used and trusted by US adults for acquiring COVID-19 information and ascertained how these sources varied according to key sociodemographic characteristics. Further, we assessed how differences in information sources were associated with variation in beliefs and levels of knowledge related to COVID-19.

## Methods

### Participant Recruitment

Details of the methods have been reported elsewhere [13]. Briefly, the sample was a self-selected nonprobability sample of social media users on Facebook and its affiliated platforms that was recruited through an on-platform advertisement campaign. Past research supports the use of Facebook as a valid, efficient, and cost-effective recruitment tool in health research [14]. The advertisement campaign targeted adults aged ≥18 years of any sex residing in the United States. Advertisements with links to an anonymous web-based survey (Qualtrics) were placed on web- or mobile-based versions of Facebook, Messenger, Instagram, and the Facebook Audience Network (other mobile apps and websites partnered with Facebook). Participants were sampled in two rounds about one month apart, from March 20 to 30 and from April 16 to 21, 2020. To reduce redundant reporting, participants could only complete the survey once (based on IP address). Eligible participants for the current study included US residents aged ≥18 years (confirmed through two screening questions). Survey reporting followed the American Association for Public Opinion Research (AAPOR) guidelines [15]. The analytic sample for this study included those with information on any of the information source variables. The New York University Institutional Review Board reviewed and exempted the study procedures, and the need for explicit written or oral consent was also waived. Participation in the study was completely voluntary and did not involve compensation, monetary or otherwise.

### Questionnaire

The survey was based on the Health Belief Model, which has been previously utilized in recent surveys on other viral outbreaks, such as H1N1 influenza [16], Middle East respiratory syndrome (MERS) [17], and Ebola [18,19]. The survey was also informed by the World Health Organization survey tool for behavioral insights on COVID-19 [20]. Sources of COVID-19–related information used by participants were measured in three ways. First, participants were asked whether or not they used any of the following sources to find information about COVID-19 (with a “not applicable” option): (1) spouse/partner, (2) other family members, (3) friends or coworkers, (4) religious leader, (5) doctor/medical provider, (6) television, (7) radio or podcasts, (8) newspaper (printed or online), (9) government or other official websites, (10) social media, and (11) Google search, Wikipedia, or other nongovernmental websites. Of the 11 variables provided, some were collapsed into categories based on shared characteristics across certain sources, with participants needing to have responded “yes” to using one or more of the listed information sources to be included in that category. The collapsed categories were the following: (1) traditional media (television, radio, podcasts, or newspapers), (2) online media (social media, Google search, Wikipedia, or other nongovernmental websites), (3) interpersonal sources (spouse/partner, other family members, and friends or coworkers). Participants were also asked if they used mainstream media sources of information for COVID-19; those who responded “yes” were asked which of the following mainstream media sources they received the most information from: (1) CNN, (2) Fox News, (3) MSNBC, (4) other local or national networks, or (5) other international networks. Second, a variable indicating the total number of sources used by each participant was created by summing the number of “yes” responses for each of the 11 information sources. Finally, participants were asked to identify the information source they trusted the most. The full questionnaire utilized in this study has been published elsewhere [13].

Knowledge and awareness of the COVID-19 outbreak and protective practices were measured by 24 binary response format (True/False) items. Examples of knowledge questions included “Coronavirus is a contagious disease” and “How can you protect yourself from being infected with coronavirus?” with options including “Getting a flu shot” and “Wearing a face mask.” Responses were consistent with information provided by the Centers for Disease Control and Prevention (CDC) as of March and April of 2020. The accuracy of the information was first assessed in March and then reassessed in April, and no changes in information accuracy were identified in this time frame. Correct responses were summed to create a composite knowledge score. Items were adapted from surveys from previous epidemics [18,21,22] and updated to reflect knowledge relevant to COVID-19.

Beliefs about COVID-19 were measured using 6 four-point (Strongly Agree, Agree, Disagree, and Strongly Disagree) Likert scale items, which were then dichotomized into a binary Agree/Disagree variable. Items were adapted from previous surveys on infectious disease outbreaks, with consideration for salient COVID-19–related beliefs at the time (March 2020) [19]. In total, three of the belief questions concerned statements on the origin, spread, or severity of COVID-19 (eg, “Coronavirus is more deadly than the seasonal flu”), for which the data were still emerging at the time of the survey, while three other questions concerned beliefs regarding the coverage and significance of the COVID-19 outbreak (eg, “The amount of media attention devoted to coronavirus has been adequate”).

Demographic variables assessed included sex, race, age category, employment status, educational attainment, living with children <18 years of age, state of residence (recoded by US Census region), urban/rural residence, and political party affiliation. Since marital status and income were only assessed in the second round of the survey and thus were missing for approximately half (55%) of the study participants, these variables were not included in the regression analyses. Participants who selected “prefer not to say” for any questions were removed from analysis, with the exception of political affiliation due to the significant number of participants selecting this option (18.9%).

### Statistical Analysis

Participant demographic characteristics were stratified by categories of information sources used. Multivariable logistic regression analyses were conducted to assess the effect of demographic determinants on the use and trust of COVID-19 information sources. Poisson regression was conducted to assess demographic determinants of the number of COVID-19 information sources given its appropriateness for modeling count data. Separate logistic regression models were conducted on the effect of time of survey (March versus April) on the use, trust, and total number of COVID-19 information sources, adjusted for demographic covariates. Of the 21 knowledge questions assessed during both rounds, 7 had a correct response rate below 90%. Logistic regression analysis was conducted on these seven questions and six COVID-19–related beliefs to assess the odds of a correct response (for knowledge questions) or of agreeing with the provided statement (for belief questions) according to the use, trust, and total number of information sources—each adjusted for the other information source variables, as well as all demographic covariates given their observed significance in health and health information seeking behaviors [23,24]. All tests were two-sided with a significance level of *P*<.05. Statistical analyses were performed using R (Version 4.0.0; R Foundation for Statistical Computing).

## Results

### Participant Characteristics

A total of 13,201 respondents were eligible to participate, of whom 12,908 commenced the survey; of these, 11,242 provided data on their sources of COVID-19 information. Due to the small sample size of participants who identified “Other” for sex (n=8), this category was unable to be analyzed and was removed for analysis. The sample size and proportion of participants who identified as races other than non-Hispanic White was small: Black, non-Hispanic (n=66, 0.6%), Asian Pacific Islander (n=86, 0.8%), Native American or American Indian (n=87, 0.8%); interracial, mixed race, or other (n=259, 2.5%); and Hispanic/Latinx (n=267, 2.6%). Therefore, in statistical analyses, participants were pooled into a singular category to enhance power in data analysis (n=765, 7.3%). Table 1 provides a summary of the participant characteristics.

**Table 1:** Characteristics of 11,242 participants with data on COVID-19 sources of information in online survey, March-April 2020^a^.

Characteristics		Source of information used						
		Total (n=11,242)	Traditional media (n=10,335)	Government (n=9845)	Online media (n=9653)	Interpersonal sources (n=7850)	Doctor (n=5361)	Religious leader (n=768)
**Time of survey (%)**								
	March	5824 (51.8)	5409 (52.3)	5333 (54.2)	5100 (52.8)	4220 (53.8)	3010 (56.1)	500 (65.1)
	April	5418 (48.2)	4926 (47.7)	4512 (45.8)	4553 (47.2)	3630 (46.2)	3196 (43.9)	268 (34.9)
**Sex (%)**								
	Female	6566 (59.0)	6124 (59.8)	5920 (60.7)	5693 (59.5)	4648 (59.7)	3196 (60.1)	293 (61.5)
	Male	4569 (41.0)	4117 (40.2)	3833 (39.3)	3868 (40.5)	3133 (40.3)	2121 (39.9)	293 (38.5)
**Age (%)**								
	18-39 years old	2360 (21.0)	2160 (20.9)	2195 (22.3)	2064 (21.4)	1878 (23.9)	1133 (21.1)	164 (21.4)
	40-59 years old	5061 (45.0)	4615 (44.7)	4479 (45.5)	4317 (44.7)	3534 (45.0)	2490 (46.4)	338 (44.0)
	≥60 years old	3821 (34.0)	3560 (34.4)	3171 (32.2)	3272 (33.9)	2438 (31.1)	1738 (32.4)	266 (34.6)
**Race (%)**								
	White, non-Hispanic	9648 (92.7)	8879 (92.7)	8449 (92.6)	8277 (92.8)	6723 (92.5)	4559 (91.6)	616 (90.9)
	Non-White	765 (7.3)	701 (7.3)	678 (7.4)	646 (7.2)	545 (7.5)	416 (8.4)	62 (9.1)
**Region (%)**								
	Northeast	2655 (26.4)	2481 (26.8)	2335 (26.4)	2282 (26.5)	1859 (26.4)	1242 (25.7)	132 (20.1)
	Midwest	2797 (27.8)	2586 (27.9)	2448 (27.7)	2380 (27.6)	1972 (28.0)	1348 (27.9)	191 (29.0)
	South	2852 (28.4)	2597 (28.0)	2513 (28.4)	2461 (28.5)	1982 (28.1)	1403 (29.0)	229 (34.8)
	West	1746 (17.4)	1597 (17.2)	1544 (17.5)	1500 (17.4)	1241 (17.6)	840 (17.4)	106 (16.1)
**Residence (%)**								
	Suburban	5126 (51.0)	4780 (51.6)	4548 (51.4)	4409 (51.1)	3622 (51.3)	2499 (51.7)	284 (43.2)
	Urban	1624 (16.2)	1496 (16.2)	1424 (16.1)	1409 (16.3)	1145 (16.2)	(786 (16.3)	90 (13.7)
	Rural	3300 (32.8)	2985 (32.2)	2868 (32.4)	2805 (32.5)	2287 (32.4)	1548 (32.0)	284 (43.2)
**Employment status (%)**								
	Employed	5980 (59.5)	5475 (59.1)	5373 (60.8)	5108 (59.2)	4397 (62.3)	2973 (61.5)	400 (60.8)
	Student or unpaid work	615 (6.1)	575 (6.2)	570 (6.4)	530 (6.1)	469 (6.6)	277 (5.7)	45 (6.8)
	Not working or unemployed	1200 (11.9)	1106 (11.9)	1043 (11.8)	1061 (12.3)	768 (10.9)	550 (11.4)	67 (10.2)
	Retired	2255 (22.4)	2105 (22.7)	1854 (21.0)	1924 (22.3)	1420 (20.1)	1033 (21.4)	146 (22.2)
**Children aged <18 years at home (%)**								
	No	7464 (71.7)	6891 (71.9)	6443 (70.6)	6379 (71.5)	5082 (69.9)	3517 (70.7)	451 (66.5)
	Yes	2949 (28.3)	2689 (28.1)	2684 (29.4)	2544 (28.5)	2186 (30.1)	1458 (29.3)	227 (33.5)
**Educational attainment (%)**								
	High school or lower	1299 (13.0)	1171 (12.7)	1100 (12.5)	1145 (13.3)	803 (11.4)	522 (10.8)	73 (11.1)
	Some college or associate degree	3428 (34.2)	3107 (33.6)	2942 (33.4)	2910 (33.8)	2332 (33.1)	1623 (33.6)	214 (32.6)
	Bachelor's degree or higher	5292 (52.8)	4957 (53.7)	4773 (54.1)	4544 (52.8)	3902 (55.4)	2679 (55.5)	370 (56.3)
**Political affiliation (%)**								
	Democrat	3609 (36.0)	3493 (37.8)	3269 (37.1)	3160 (36.7)	2659 (37.8)	1870 (38.8)	165 (25.1)
	Republican	2503 (25.0)	2227 (24.1)	2106 (23.9)	2134 (24.8)	1706 (24.2)	1084 (22.5)	256 (39.0)
	Other	2009 (20.1)	1807 (19.6)	1769 (20.1)	1703 (19.8)	1374 (19.5)	970 (20.1)	94 (14.3)
	Prefer not to say	1898 (18.9)	1708 (18.5)	1671 (19.0)	1602 (18.6)	1298 (18.4)	900 (18.7)	142 (21.6)
**Marital status (%)**								
	Married or cohabiting	3583 (70.9)	3269 (71.0)	3023 (71.6)	3001 (70.6)	2538 (74.3)	1616 (73.2)	185 (74.6)
	Single	830 (16.4)	754 (16.4)	688 (16.3)	711 (16.7)	527 (15.4)	345 (15.6)	34 (13.7)
	Divorced or separated	429 (8.5)	389 (8.4)	348 (8.2)	370 (8.7)	233 (6.8)	163 (7.4)	19 (7.7)
	Widowed	213 (4.2)	195 (4.2)	166 (3.9)	171 (4.0)	118 (3.5)	85 (3.8)	10 (4.0)
**Income (%)**								
	<$30,000	577 (13.3)	515 (13.0)	462 (12.7)	487 (13.3)	330 (11.3)	228 (11.9)	32 (15.5)
	$30,000 to less than $50,000	671 (15.5)	616 (15.6)	567 (15.5)	576 (15.8)	437 (15.0)	271 (14.2)	22 (14.0)
	$50,000 to less than $75,000	767 (17.7)	687 (17.4)	618 (16.9)	636 (17.4)	484 (16.6)	331 (17.3)	46 (22.2)
	$75,000 to less than $100,000	900 (20.8)	831 (21.0)	764 (20.9)	749 (20.5)	616 (21.2)	424 (22.1)	44 (21.3)
	≥$100,000	1418 (32.7)	1310 (33.1)	1237 (33.9)	1202 (32.9)	1044 (35.9)	661 (34.5)	56 (27.1)
**Most trusted source (%)**								
	Government or other official websites	4867 (45.2)	4497 (45.4)	4673 (49.4)	4109 (44.4)	3297 (43.8)	2123 (41.2)	310 (42.9)
	Television	509 (4.7)	503 (5.1)	362 (3.8)	458 (5.0)	330 (4.4)	132 (2.6)	22 (3.0)
	Social media	131 (1.2)	105 (1.1)	81 (0.9)	126 (1.4)	82 (1.1)	29 (0.6)	10 (1.4)
	Newspaper	699 (6.5)	691 (7.0)	599 (6.3)	636 (6.9)	523 (6.9)	270 (5.2)	35 (4.8)
	Other web-based sources	549 (5.1)	459 (4.6)	423 (4.5)	527 (5.7)	345 (4.6)	152 (3.0)	22 (3.0)
	Friends or coworkers	49 (0.5)	37 (0.4)	34 (0.4)	40 (0.4)	43 (0.6)	18 (0.3)	3 (0.4)
	Doctor or medical provider	3408 (31.6)	3134 (31.6)	2909 (30.8)	2891 (31.3)	2444 (32.4)	2239 (43.5)	259 (35.9)
	Radio or podcasts	115 (1.1)	114 (1.2)	81 (0.9)	101 (1.1)	75 (1.0)	39 (0.8)	12 (1.7)
	Other family members	118 (1.1)	106 (1.1)	76 (0.8)	106 (1.1)	107 (1.4)	41 (0.8)	32 (1.4)
	Spouse or partner	316 (2.9)	261 (2.6)	214 (2.3)	247 (2.7)	279 (3.7)	102 (2.0)	10 (4.4)
	Religious leader	8 (0.1)	6 (0.1)	6 (0.1)	7 (0.1)	7 (0.1)	3 (0.1)	7 (1.0)

^a^Total number of responses with data on sources of information, excluding those selecting “not applicable” for all sources.

**Figure 1:**
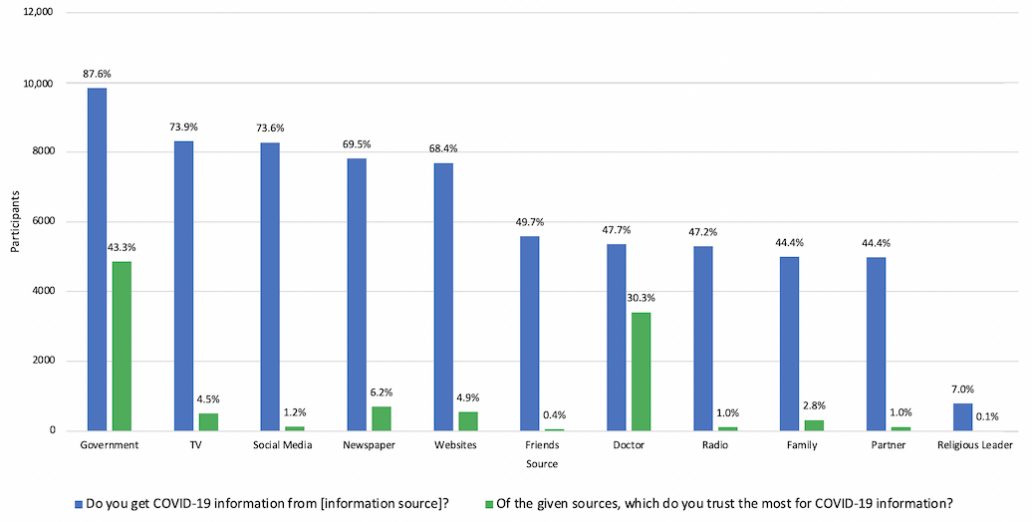
Distribution of sources used and most trusted sources for information on COVID-19 (N=11,242).

Geographic representation of participants included all US states. Overall, most participants were female (59.0%), non-Hispanic white (92.7%), employed (59.5%), and living in suburban environments (51.0%). Figure 1 displays an overview of the information sources used and most trusted by the study population. Overall, traditional media was the most frequently utilized source of information (n=10,335, 91.2%); however, when all information sources were disaggregated from the synthesized categories, the largest individual source of COVID-19 information was government websites (n=9845, 87.6%). Participants used an average of 6.1 sources (SD 2.3, range 0-11). Among those who used mainstream media sources for COVID-19 information (n=7811, 69.5%), other local or national networks were the most popular sources of COVID-19 information (35.2%), followed by CNN (24.0%), Fox News (19.3%), MSNBC (11.9%), and other international networks (5.3%).

### Sociodemographic Factors Associated With Sources of COVID-19 Information

Males were significantly less likely than females to use all identified sources, excluding spouses/family/friends and religious leaders (Table 2). Participants aged 40-59 years and ≥60 years were less likely to use government websites compared to those aged 18-38 years (adjusted odds ratio [AOR] 0.59, 95% CI 0.47-0.71; AOR 0.47, 95% CI 0.37-0.60). Participants identifying as races other than non-Hispanic White were more likely to use doctors (AOR 1.39, 95% CI 1.18-1.64) and religious leaders (AOR 1.40, 95% CI 1.03-1.86) as a source of information. Those with a bachelor’s degree or higher were more likely to use all of the sources, except traditional media.

Sociodemographic predictors of using mainstream media sources for COVID-19 information are displayed in Multimedia Appendix 1. Republicans were significantly more likely to rely upon Fox News (AOR 33.56, 95% CI 25.60-44.87), while they were less likely to rely on all other mainstream media sources. In contrast, those with a bachelor’s degree or higher were more likely to rely on CNN (AOR 1.25, 95% CI 1.04-1.52) or other international networks (AOR 3.68, 95% CI 2.21-6.68) and less likely to rely on Fox News (AOR 0.72, 95% CI 0.61-0.87). Participants aged ≥60 years were more likely to rely on Fox News (AOR 1.41, 95% CI 1.12-1.77) and MSNBC (AOR 1.85, 95% CI 1.43-2.40) and less likely to rely on other international sources (AOR 0.67, 95% CI 0.47-0.95).

**Table 2:** Adjusted odds ratios (95% CI) of sociodemographic factors associated with COVID-19 information source (N=11,242)^a^. Ref: Reference group.

Sociodemographic factors			Traditional media		Government		Online media		Interpersonal sources		Doctor		Religious leader
**Sex**													
	Female	Ref		Ref		Ref		Ref		Ref		Ref	
	Male	0.74 (0.64-0.87)^b^		0.58 (0.51-0.66)^b^		0.88 (0.78-0.99)^d^		0.96 (0.88-1.06)		0.91 (0.84-0.99)^d^		0.89 (0.75-1.06)	
**Age**													
	18-39 years	Ref		Ref		Ref		Ref		Ref		Ref	
	40-59 years	0.99 (0.80-1.21)		0.58 (0.47-0.71)^b^		0.80 (0.68-0.94)^c^		0.61 (0.54-0.70)^b^		1.10 (0.96-1.23)		0.86 (0.69-1.08)	
	≥60 years	1.12 (0.85-1.46)		0.47 (0.37-0.60)^b^		0.91 (0.74-1.11)		0.52 (0.45-0.62)^b^		1.02 (0.88-1.17)		1.07 (0.81-1.42)	
**Race**													
	White	Ref		Ref		Ref		Ref		Ref		Ref	
	Non-White	0.96 (0.72-1.29)		0.95 (0.74-1.23)		0.87 (0.71-1.09)		1.02 (0.85-1.22)		1.39 (1.18-1.64)^b^		1.40 (1.03-1.86)^d^	
**Region**													
	Northeast	Ref		Ref		Ref		Ref		Ref		Ref	
	Midwest	0.88 (0.71-1.09)		0.95 (0.80-1.13)		0.94 (0.71-1.09)		0.98 (0.87-1.11)		1.07 (0.96-1.20)		1.30 (1.03-1.65)^d^	
	South	0.74 (0.60-0.92)^c^		1.09 (0.92-1.29)		1.06 (0.90-1.23)		0.97 (0.86-1.09)		1.10 (0.98-1.23)		1.50 (1.20-1.89)^b^	
	West	0.69 (0.55-0.88)^c^		1.07 (0.88-1.30)		1.00 (0.84-1.19)		1.03 (0.89-1.18)		1.04 (0.91-1.18)		1.28 (0.97-1.67)	
**Residence**													
	Suburban	Ref		Ref		Ref		Ref		Ref		Ref	
	Urban	0.81 (0.65-1.01)		0.95 (0.79-1.14)		1.05 (0.90-1.23)		1.02 (0.89-1.16)		1.00 (0.89-1.13)		1.28 (0.97-1.67)	
	Rural	0.80 (0.67-0.94)^c^		1.01 (0.88-1.17)		0.94 (0.83-1.25)		1.07 (0.96-1.18)		0.99 (0.90-1.09)		1.56 (1.31-1.86)^b^	
**Employment**													
	Employed	Ref		Ref		Ref		Ref		Ref		Ref	
	Student or unpaid	1.26 (0.89-1.83)		0.99 (0.71-1.39)		0.97 (0.76-1.25)		0.97 (0.79-1.20)		0.81 (0.68-0.97)^d^		1.05 (0.74-1.47)	
	Not working or unemployed	1.11 (0.87-1.42)		0.79 (0.65-0.96)^d^		1.30 (1.07-1.59)^c^		0.70 (0.61-0.80)^b^		0.87 (0.76-0.99)^d^		0.94 (0.71-1.23)	
	Retired	1.16 (0.91-1.48)		0.68 (0.57-0.82)^b^		0.96 (0.81-1.15)		0.78 (0.68-0.89)^b^		0.90 (0.79-1.03)		0.91 (0.71-1.16)	
**Children at home**													
	No	Ref		Ref		Ref		Ref		Ref		Ref	
	Yes	0.94 (0.78-1.12)		1.18 (1.00-1.39)		1.04 (0.91-1.19)		1.05 (0.94-1.17)		1.06 (0.96-1.17)		1.25 (1.03-1.51)^d^	
**Education**													
	High school or lower	Ref		Ref		Ref		Ref		Ref		Ref	
	Some college or associate degree	0.94 (0.74-1.17)		1.09 (0.90-1.31)		0.75 (0.62-0.91)^c^		1.28 (1.12-1.48)^b^		1.29 (1.13-1.48)^b^		1.21 (0.92-1.61)	
	Bachelor’s degree or higher	1.23 (0.98-1.55)		1.49 (1.23-1.79)^b^		0.78 (0.64-0.94)^d^		1.62 (1.42-1.86)^b^		1.39 (1.22-1.59)^b^		1.56 (1.20-2.06)^c^	
**Political affiliation**													
	Democrat	Ref		Ref		Ref		Ref		Ref		Ref	
	Republican	0.30 (0.23-0.38)^b^		0.63 (0.53-0.74)^b^		0.86 (0.73-1.00)		0.83 (0.74-0.94)^c^		0.71 (0.64-0.80)^b^		2.24 (1.81-2.79)^b^	
	Other	0.32 (0.25-0.41)^b^		0.79 (0.66-0.96)^d^		0.80 (0.68-0.94)^c^		0.77 (0.67-0.87)^b^		0.90 (0.80-1.01)		1.03 (0.79-1.34)	
	Prefer not to say	0.31 (0.24-0.40)^b^		0.80 (0.66-0.96)^d^		0.78 (0.66-0.92)^c^		0.83 (0.73-0.94)^c^		0.90 (0.80-1.01)^c^		1.71 (1.35-2.18)^b^	

^a^Odds of using source compared to those not using source, adjusting for all other covariates in tables.

^b^*P*<.001.

^c^*P*<.01.

^d^*P*<.05.

With respect to predictors of the total number of COVID-19 sources used (not shown in tables), those using fewer sources included males compared to females (β=–0.03, 95% CI –0.04 to –0.01) and those aged 40-59 years and ≥60 years compared to those aged 18-39 years (β=–0.05, 95% CI –0.07 to –0.03; β=–0.05, 95% CI –0.08 to –0.03). Factors associated with increased number of sources included having children in the home compared to not having children in the home (β=0.03, 95% CI 0.01-0.05), and having some college or a bachelor’s degree or a higher level of educational attainment compared to those with a high school diploma or less educational attainment (β=0.03, 95% CI 0.00-0.06; β=0.08, 95% CI 0.05-0.10).

The most trusted information source was government websites (45.2%). The odds of trusting government websites were lower among males (AOR 0.58, 95% CI 0.53-0.63) and those aged 40-59 years and ≥60 years compared to those aged 18-38 years (AOR 0.83, 95% CI 0.74-0.92; AOR 0.62, 95% CI 0.54-0.71; data not shown in tables).

Overall, participants were significantly less likely to use any of the identified information sources in April compared to March (Multimedia Appendix 2); the adjusted odds of using government websites in April compared to March were particularly low (AOR 0.41, 95% CI 0.36-0.47). Similarly, compared to March, the odds of trusting government websites in April were significantly lower (AOR 0.51, 95% CI 0.47-0.56), while the odds of trusting other websites, radios or podcasts, and spouses/partners more than doubling during that same time frame. In addition, participants in April used on average 0.58 fewer sources than those in March (*P*<.001).

The adjusted associations between COVID-19 information sources and knowledge of COVID-19 varied considerably by knowledge question (Table 3). An increase in the total number of information sources used was only associated with improved awareness that wearing a face mask was protective against COVID-19 infection (AOR 1.10, 95% CI 1.05-1.14). The use of some information sources, such as doctors and traditional media, were associated with improved knowledge for some questions but decreased knowledge for others. Overall, the use of government websites resulted in significantly better knowledge for 3 of the 7 examined questions, with the remaining 4 questions not significantly different between the groups.

The primary mainstream media source used for COVID-19 information was also significantly associated with knowledge about the pandemic (not shown in table). When adjusted for sociodemographic variables, total number of sources, and the most trusted source of information, those relying on CNN were more likely than those relying on other local/national media sources to correctly answer 2 of the 7 questions, while those relying on Fox News were more likely to incorrectly answer 3 of the 7 questions.

**Table 3:** Adjusted odds ratios (95% CI) of COVID-19 knowledge (correct answer) by information source (N=11,242)^a^. Ref: Reference group.

Sources		Currently, there is an FDA-approved drug for treating individuals with the coronavirus.	Children are at high risk for complications from the coronavirus.	Alcohol-based hand sanitizers cannot protect you from the coronavirus.	The coronavirus originated from animals.	How can you protect against the coronavirus infection? Getting a flu shot.	How can you protect against the coronavirus infection? Wearing a face mask.	How can you protect against the coronavirus infection? Stop going to school/work.
Number of sources		1.03 (0.97-1.10)	1.03 (0.98-1.08)	1.04 (0.98-1.09)	0.96 (0.92-1.02)	0.97 (0.92-1.02)	1.10 (1.05-1.14)^b^	1.07 (1.00-1.14)^d^
**Source group**								
	Traditional media	1.24 (0.95-1.61)	0.93 (0.72-1.19)	1.28 (1.00-1.63)^d^	0.78 (0.61-1.00)^d^	1.09 (0.83-1.40)	1.45 (1.18-1.77)^b^	1.93 (1.50-2.48)^b^
	Government	1.30 (1.04-1.61)^d^	1.21 (0.99-1.46)	1.12 (0.91-1.36)	0.90 (0.73-1.10)	1.33 (1.08-1.62)^c^	0.88 (0.74-1.05)	1.44 (1.16-1.79)^c^
	Online media	1.07 (0.85-1.35)	1.03 (0.84-1.25)	0.92 (0.74-1.13)	1.09 (0.89-1.34)	1.06 (0.86-1.30)	0.96 (0.82-1.13)	1.34 (1.06-1.68)^d^
	Interpersonal sources	1.00 (0.81-1.22)	0.91 (0.77-1.08)	0.99 (0.83-1.18)	1.01 (0.85-1.20)	1.08 (0.91-1.29)	0.98 (0.85-1.12)	1.16 (0.94-1.42)
	Doctor	0.91 (0.77-1.07)	0.82 (0.72-0.94)^c^	0.99 (0.86-1.15)	1.01 (0.87-1.16)	0.88 (0.76-1.02)	1.17 (1.05-1.31)^c^	1.09 (0.92-1.31)
	Religious leader	0.65 (0.49-0.86)^c^	0.86 (0.68-1.10)	1.09 (0.83-1.44)	0.92 (0.69-1.21)	0.91 (0.71-1.17)	0.81 (0.67-0.99)^d^	1.02 (0.72-1.45)
**Most trusted source^e^**								
	Government	Ref	Ref	Ref	Ref	Ref	Ref	Ref
	Television	0.54 (0.40-0.75)^b^	0.83 (0.63-1.09)	0.85 (0.64-1.14)	1.18 (0.88-1.55)	0.82 (0.62-1.09)	1.38 (1.08-1.79)^d^	0.81 (0.55-1.22)
	Social media	0.43 (0.26-0.73)^c^	1.12 (0.65-2.07)	0.67 (0.41-1.17)	1.48 (0.85-2.46)	1.46 (0.78-3.04)	0.77 (0.49-1.23)	0.29 (0.17-0.49)^b^
	Newspaper	1.40 (0.92-2.22)	1.11 (0.84-1.47)	1.32 (0.98-1.79)	0.76 (0.56-1.02)	1.17 (0.88-1.58)	0.97 (0.78-1.20)	0.98 (0.66-1.49)
	Websites	0.54 (0.40-0.72)^b^	1.12 (0.84-1.53)	0.89 (0.67-1.18)	1.13 (0.84-1.49)	1.67 (1.18-2.42)^c^	0.67 (0.53-0.84)^c^	0.32 (0.24-0.42)^b^
	Friends	0.41 (0.19-1.00)^d^	0.81 (0.37-2.06)	1.96 (0.70-8.20)	0.51 (0.12-1.44)	1.00 (0.42-2.97)	0.80 (0.40-1.66)	0.25 (0.11-0.56)^b^
	Doctor	0.84 (0.71-1.00)^d^	0.88 (0.77-1.01)	0.97 (0.84-1.13)	1.03 (0.88-1.19)	0.81 (0.70-0.93)^c^	1.05 (0.94-1.18)	0.63 (0.52-0.75)^b^
	Radio	0.42 (0.25-0.73)^c^	1.34 (0.73-2.72)	0.46 (0.28-0.77)^c^	2.19 (1.30-3.58)^c^	0.83 (0.48-1.56)	0.82 (0.51-1.37)	0.28 (0.17-0.47)^b^
	Partner	0.60 (0.42-0.86)^c^	1.01 (0.72-1.46)	0.71 (0.50-1.01)^d^	1.42 (1.00-1.99)^d^	0.83 (0.59-1.21)	0.89 (0.66-1.20)	0.33 (0.23-0.47)^b^
	Family	0.77 (0.42-1.54)	0.68 (0.41-1.19)	0.96 (0.55-1.81)	1.04 (0.55-1.83)	0.65 (0.38-1.14)	0.85 (0.53-1.40)	0.49 (0.27-0.95)^d^

^a^Adjusted for all other information source variables in the model, as well as time of survey, sex, age, race, region, type of residence, working status, children, education, and political affiliation.

^b^*P*<.001.

^c^*P*<.01.

^d^*P*<.05.

^e^Due to the small sample size of those identifying religious leaders as their most trusted source (n=8), these were removed for analysis.

Changes in beliefs regarding COVID-19 were observed to be strongly and consistently associated with both the use of and trust in different information sources (Table 4). Compared to participants that did not use government websites, those who used government websites were more likely to disagree with the following statements: the coronavirus was released as an act of terrorism (AOR 0.64, 95% CI 0.54-0.76), the coronavirus is not as big a problem as the media suggests (AOR 0.65, 95% CI 0.53-0.78), and warmer weather will reduce the spread of the coronavirus (AOR 0.69, 95% CI 0.58-0.80). Compared to those with the most trust in government websites, trust in most (≥6) of the other sources of information was associated with increased agreement that the coronavirus was released as an act of terrorism, disagreement that the coronavirus is deadlier than the flu, agreement that the coronavirus is not as big a problem as the media suggests, and disagreement that the amount of media attention on the coronavirus has been adequate. Mainstream media source was also a significant determinant for COVID-19 beliefs (not shown in tables). Compared to those relying on other national/local media, those relying on CNN or MSNBC were more likely to agree that the coronavirus is deadlier than the seasonal flu, the amount of media attention devoted to the coronavirus has been adequate, and the coronavirus is a bigger problem than the government suggests. In addition, they were more likely to disagree that warmer weather will reduce the spread of the coronavirus and that the coronavirus is not as big of a problem as the media suggests. Conversely, those relying on Fox News were more likely to agree that the coronavirus was released as an act of bioterrorism, warmer weather will reduce the spread of the coronavirus, and the coronavirus is not as big of a problem as the media suggests. In addition, they were more likely to disagree that the coronavirus is deadlier than the seasonal flu, the amount of media attention devoted to the coronavirus has been adequate, and the coronavirus is a bigger problem than the government suggests.

**Table 4:** Adjusted odds ratios (95% CI) of agreement of COVID-19 beliefs by information source, n=11,242^a^. Ref: Reference group.

Sources		I think that the coronavirus was released as an act of bioterrorism.	The coronavirus is more deadly than the seasonal flu.	I think warmer weather will reduce the spread of the coronavirus.	The amount of media attention devoted to the coronavirus has been adequate.	The coronavirus is not as big of a problem as the media suggests.	The coronavirus is a bigger problem than the government suggests.
	Number of sources	1.02 (0.97-1.06)	1.13 (1.08-1.19)^b^	1.02 (0.98-1.06)	1.13 (1.07-1.19)^b^	0.91 (0.86-0.95)^b^	1.08 (1.03-1.12)^c^
**Source group**							
	Traditional media	0.51 (0.42-0.64)^b^	1.60 (1.29-1.98)^b^	0.76 (0.63-0.92)^c^	1.54 (1.24-1.19)^b^	0.47 (0.37-0.58)^b^	1.53 (1.24-1.89)^b^
	Government	0.64 (0.54-0.76)^b^	1.48 (1.23-1.77)^b^	0.69 (0.58-0.80)^b^	1.10 (0.90-1.32)	0.65 (0.53-0.78)^b^	1.25 (1.05-1.48)^d^
	Online media	0.98 (0.81-1.17)	1.09 (0.90-1.31)	1.07 (0.92-1.25)	1.05 (0.87-1.27)	1.05 (0.87-1.27)	1.03 (0.87-1.21)
	Interpersonal sources	0.89 (0.76-1.04)	0.96 (0.82-1.13)	0.97 (0.85-1.11)	0.81 (0.69-0.96)^d^	0.90 (0.77-1.06)	0.95 (0.82-1.09)
	Doctor	0.85 (0.75-0.96)^d^	1.02 (0.89-1.17)	0.84 (0.75-0.93)^c^	0.97 (0.85-1.12)	0.84 (0.73-0.96)^c^	1.24 (1.10-1.39)^b^
	Religious leader	1.38 (1.11-1.70)^c^	0.78 (0.61-1.00)^d^	1.36 (1.12-1.64)^c^	0.74 (0.58-0.95)^d^	1.58 (1.25-1.99)^b^	0.57 (0.47-0.70)^b^
**Most trusted source^e^**							
	Government	Ref	Ref	Ref	Ref	Ref	Ref
	Television	1.53 (1.19-1.97)^c^	0.96 (0.71-1.32)	0.95 (0.75-1.19)	1.55 (1.10-2.25)^d^	1.00 (0.74-1.43)	1.34 (1.04-1.74)^d^
	Social media	2.52 (1.58-4.03)^b^	0.50 (0.31-0.81)^d^	1.99 (1.25-3.26)^c^	0.50 (0.32-0.81)^c^	2.46 (1.48-4.14)^c^	0.83 (0.51-1.35)
	Newspaper	0.66 (0.48-0.90)^c^	1.07 (0.78-1.48)	0.89 (0.73-1.09)	1.67 (1.21-2.36)^c^	0.86 (0.63-1.15)	1.44 (1.13-1.84)^c^
	Websites	2.08 (1.65-2.62)^b^	0.43 (0.34-0.55)^b^	1.37 (1.10-1.70)^c^	0.64 (0.50-0.82)^b^	2.27 (1.78-2.89)^b^	0.60 (0.48-0.76)^b^
	Friends	3.01 (1.48-6.08)^c^	0.65 (0.30-1.49)	1.43 (0.73-2.86)	0.42 (0.21-0.88)^d^	2.58 (1.18-5.58)^d^	0.52 (0.24-1.07)
	Doctor	1.45 (1.27-1.64)^b^	0.70 (0.61-0.81)^b^	1.07 (0.96-1.20)	0.82 (0.71-0.94)^c^	1.37 (1.20-1.57)^b^	0.97 (0.86-1.10)
	Radio	1.55 (0.95-2.51)	0.37 (0.23-0.61)^b^	2.15 (1.34-3.53)^c^	0.72 (0.44-1.24)	3.16 (1.88-5.38)^b^	0.54 (0.32-0.88)^d^
	Partner	2.82 (2.09-3.81)^b^	0.48 (0.36-0.66)^b^	1.73 (1.30-2.33)^b^	0.60 (0.45-0.82)^c^	2.07 (1.49-2.89)^b^	0.66 (0.48-0.89)^c^
	Family	3.78 (2.36-6.11)^b^	0.59 (0.36-1.00)^d^	1.31 (0.83-2.07)	0.62 (0.37-1.05)	1.56 (0.92-2.59)	1.00 (0.62-1.63)

^a^Adjusted for all other information source variables in the model, as well as time of survey, sex, age, race, region, type of residence, working status, children, education, political affiliation.

^b^*P*<.001.

^c^*P*<.01.

^d^*P*<.05.

^e^Due to the small sample size of those identifying religious leaders as their most trusted source (n=8), these were removed for analysis.

## Discussion

### Principal Findings

Overall, the choice of and trust in different COVID-19 information sources were observed to be significantly different across demographic variables, including sex, age, race, region, residence type, employment, education, and political affiliation. In addition, the type of source used and trust in each source were associated with different levels of knowledge and differences in beliefs regarding COVID-19. Despite advocacy by public health officials for the use of official or government sources of information (such as CDC or World Health Organization websites), only 45.2% of participants cited such sources as their most trusted sources of information, dropping from 53.3% in March to 36.8% in April. These findings suggest that public health professionals seeking to effectively communicate information on COVID-19 must acknowledge and appropriately adapt to disparities in public trust and information source preferences, particularly to address the differences in knowledge and beliefs regarding the pandemic.

### Popular and Trusted Information Sources

The popularity of television and newspapers as sources of information during the current outbreak reflected past infectious disease outbreaks including the 2003 severe acute respiratory syndrome (SARS) outbreak [8], the 2009 H1N1 pandemic [9,10], and seasonal flu epidemics [11]. A qualitative study on communication during pandemics found that mainstream media (such as newspapers and television) were the most used source of information among participants despite being perceived as relatively untrustworthy [4], corroborating our study findings. However, while our study found government websites and doctors to be the most trusted sources, a study of the SARS outbreak in the Netherlands found that television and health officials were the most trusted sources of information [8], while a study of Ebola information in the United States found the internet and the government to be the most trusted information sources [12].

Findings that men were less likely to use almost all of the identified information sources, used fewer sources in general, and were also less likely to trust government websites for COVID-19 information suggest significant sex disparities in COVID-19 information source utilization. This evidence corresponds with other preliminary research observing that men are less likely to abide by advocated COVID-19 health behaviors [25], and that young men are more likely to agree with COVID-19 myths [26] and underscores the need for a sex-based targeted COVID-19 health information communication strategy. Moreover, age- and education-based disparities in COVID-19 knowledge and behaviors have also been observed in past research [27]. Given these disparities in COVID-19 information source usage, there is a clear need for targeted health communication campaigns to address these gaps. Lastly, disparities based on political affiliation also correspond with other evidence of its potential role in determining COVID-19 behaviors, including compliance with social distancing [28]. The use of specific mainstream media outlets was found to be significantly determined by political affiliation, sex, and age, determinants which support past demographic analyses on mainstream media usage [29].

These findings suggest that trust in information sources may differ across time, place, culture, and type of disease outbreak, emphasizing the importance of updated surveillance on trends in information seeking behaviors during pandemics. For instance, the greater popularity of and trust in government sources may be explained by strong efforts by nongovernmental platforms such as Facebook, Google, and Twitter to promote official government websites [30]. Moreover, due to the small sample size of participants who identified as races other than non-Hispanic White, we were unable to conduct a comprehensive analysis on disaggregated racial and ethnic differences. However, past research has found immigrant communities from Asia have also been observed to display less confidence in their doctors and government agencies compared to other populations [31], suggesting a need for further intensive research on COVID-19 information source trends among minority communities in the United States, especially given their higher risk for morbidity and mortality from COVID-19 [32,33].

Likewise, unlike past research, the uniquely imminent and personal threat posed to members of the public responding to the survey should be considered. In other words, the Netherlands did not experience a SARS epidemic, nor did the United States experience an Ebola epidemic, and comparing perceptions regarding which information sources to consult and trust with these past case studies may differ depending on the level of perceived and actual risk within the population [34]. To meet the health communication needs of future pandemics or public health crises such as COVID-19, public health professionals and policy makers must conduct careful monitoring on up-to-date trends in information source usage to better target the delivery of public health information.

Importantly, findings also suggest that the likelihood of using different COVID-19 information sources changed between March and April. The finding that participants were less likely to trust sources such as government websites in this time frame has significant implications on the speed with which targeted public health information campaigns may need to be implemented to meet these rapid changes in information source utilization. Indeed, similar preliminary research has also observed a decline in trust of COVID-19 information provided by government sources [35]. Such evidence provides insight into how perceptions and utilization of information sources may significantly vary across different stages of a health crisis, supporting the need for continued, longitudinal public health surveillance to help the relevant authorities understand these trends and take action accordingly.

### COVID-19 Knowledge and Information Sources

There have been concerns about the surge and spread of dangerous misinformation related to COVID-19 [36], including through online platforms like social media [37], particularly because there are many aspects of this novel disease that are presently poorly understood or subject to change as new evidence becomes available. Likewise, mainstream media sources have also garnered greater scrutiny over concerns that COVID-19 misinformation is being perpetuated by certain media outlets [38], which was supported by the disparities in knowledge observed across those relying on different mainstream media sources. However, our findings also suggested that the use of and trust in information sources other than official government websites may not be associated with significantly different awareness of information about an emerging health crisis such as COVID-19. For instance, while social media has garnered increased attention due to its use as a platform to promulgate COVID-19 misinformation [37], the use of social media or web-based sources was in fact associated with increased awareness of one of the seven knowledge questions and had no effect on the other questions. Furthermore, those who trusted social media information the most (compared to government websites) only displayed reduced knowledge for two questions. Moreover, the sources from which members of the public obtain COVID-19 information may be interdependent; in previous influenza pandemics, doctors (who are a major source of health information) reported deriving much of their information from the internet and mass media [39,40]. These insights suggest that certain sources of information may not inherently result in compromised awareness of information pertaining to a health crisis, but other factors, such as the actual content, how the source is used by an individual, and the specific knowledge being assessed, may all play a relevant role in determining disparities in knowledge.

### COVID-19 Beliefs and Information Source

Unlike with knowledge outcomes, the strong associations observed between both trust in and use of different information sources and COVID-19 beliefs suggest that different communication platforms are indeed having an impact on how the public subjectively perceives and interprets COVID-19 information. These associations were also observed in the reliance on different types of mainstream media sources, suggesting specific media outlets may also have a salient role in perpetuating certain beliefs about the pandemic. The perception that the coronavirus is deadlier than the flu was significantly higher among those who used and put the greatest trust in government websites, suggesting that these platforms have been able to effectively communicate the relative greater danger of COVID-19. Beliefs regarding communication of COVID-19 information displayed similar trends, with individuals trusting of different nongovernmental sources expressing greater agreement that the coronavirus is not as serious a problem as the media suggests, and disagreement that the coronavirus is a bigger problem than the government suggests. In a review on attitudes and beliefs during pandemics, various subjective understandings of the spread and significance of an infectious disease were directly associated with protective behaviors [41], suggesting that differing levels of seriousness and perceived significance of a pandemic can have consequences for the collective public response.

### Strengths and Limitations

This study was subject to a number of key limitations. First, the study sample was derived from nonprobability convenience sampling of Facebook and affiliated platform users, and although 70% of Americans use Facebook [5], certain demographic groups may be underrepresented (eg, racial, ethnic, and gender minorities), which is one component of why national representativeness cannot be assumed. Second, many of the information sources categorized are themselves inconsistent, such that within each source, the types and veracity of information vary markedly, and these disparities must be considered in interpreting the study findings. For example, given the wide variety of internet-based information sources, it is likely that many nonofficial or nongovernmental websites are providing valid, up-to-date information on COVID-19 and thus correlations between knowledge and beliefs about the pandemic may be significantly dependent on the specific internet sources being utilized rather than simply the platform itself, as observed in other health contexts [42]. Likewise, in recent years, other information source categories (such as social media) have significantly diversified (eg, web-based and app-based platforms, or those more video-based platforms such as TikTok), and this internal diversity may also influence trends in COVID-19 information source. Therefore, further research targeting more specific, stratified sources of information is warranted. Finally, given the emerging nature of the COVID-19 crisis, knowledge and salient beliefs are constantly evolving, and while the survey reflects a number of key questions relevant during March and April 2020, many of these may not be relevant in future months or years of the crisis. To address this, the survey used in the study will be adapted and reimplemented periodically over the course of the COVID crisis.

### Conclusion

As the need to rapidly communicate information about the ongoing COVID-19 pandemic persists, our study findings provide key insights to policy makers seeking to understand what impact these information seeking behaviors are having on knowledge and beliefs regarding the outbreak. Likewise, information on the demographic profiles of who is using and trusting different information sources allows public health professionals to adapt communication strategies to reach a more diverse population. Future research should consider greater sampling of minority populations in the United States (notably racial and ethnic minorities, non-English speakers, and non-internet users) to provide further perspective on disparities in information seeking behaviors during COVID-19 and other health crises.

## Appendix

Multimedia Appendix 1 Sociodemographic factors associated with mainstream media sources of COVID-19 information.

Multimedia Appendix 2 Changes in COVID-19 information source between March and April 2020.

## Abbreviations

CDC: Centers for Disease Control and Prevention
