# Supplementary material for: Trends and Predictors of COVID-19 Information Sources and Their Relationship With Knowledge and Beliefs Related to the Pandemic: Nationwide Cross-Sectional Study
Source: JMIR Public Health Surveill. 2020 Oct 8;6(4):e21071. doi: 10.2196/21071 (PMC7546863; doi:10.2196/21071)
Supplement: Multimedia Appendix 1 [file publichealth_v6i4e21071_app1.docx]

**Table 1. Socio-demographic factors associated with mainstream media sources of COVID-19 information source^, n = 7,811**

|  | *CNN* | *Fox News* | *MSNBC* | *Other national/local* | *Other international* |
| --- | --- | --- | --- | --- | --- |
| **Sex** |  |  |  |  |  |
| Female | Ref | Ref | Ref | Ref | Ref |
| Male | 0.95 (0.84-1.06) | 1.05 (0.92-1.19) | 0.96 (0.82-1.12) | **^3^ 0.69 (0.63-0.76)** | 1.08 (0.87-1.34) |
| **Age** |  |  |  |  |  |
| 18-39 years | Ref | Ref | Ref | Ref | Ref |
| 40-59 years | 1.00 (0.87-1.15) | 1.18 (0.98-1.43) | 1.16 (0.93-1.46) | 1.10 (0.97-1.25) | 0.89 (0.69-1.16) |
| 60+ years | 0.87 (072-1.05) | **^2^ 1.41 (1.12-1.77)** | **^3^ 1.85 (1.43-2.40)** | 1.11 (0.95-1.30) | **^2^ 0.67 (0.47-0.95)** |
| **Race** |  |  |  |  |  |
| White | Ref | Ref | Ref | Ref | Ref |
| Non-White | 0.97 (0.79-1.19) | 0.88 (0.66-1.15) | 0.82 (0.60-1.11) | 0.98 (0.82-1.17) | **^3^ 1.44 (1.03-1.97)** |
| **Region** |  |  |  |  |  |
| Northeast | Ref | Ref | Ref | Ref | Ref |
| Midwest | 0.87 (0.75-1.01) | 0.99 (0.83-1.17) | 0.95 (0.73-1.15) | 0.93 (0.83-1.06) | 1.11 (0.82-1.51) |
| South | 1.13 (0.98-1.30) | 1.00 (0.85-1.18) | 0.85 (0.69-1.03) | **^3^ 0.78 (0.69-0.88)** | 1.30 (0.97-1.75) |
| West | **^2^ 0.79 (0.67-0.93)** | **^1^ 0.80 (0.65-0.99)** | **^3^ 0.79 (0.63-0.98)** | 0.89 (0.77-1.02) | **^2^ 1.55 (1.14-2.11)** |
| **Residence** |  |  |  |  |  |
| Suburb. | Ref | Ref | Ref | Ref | Ref |
| Urban | 1.06 (0.92-1.23) | **^1^ 0.79 (0.64-0.99)** | 1.08 (0.88-1.31) | 0.95 (0.83-1.08) | **^1^ 1.38 (1.06-1.78)** |
| Rural | **^2^ 0.83 (0.73-0.94)** | **^2^ 0.81 (0.71-0.93)** | 0.99 (0.84-1.18) | 0.99 (0.89-1.10) | 0.92 (0.71-1.19) |
| **Employment** |  |  |  |  |  |
| Employed | Ref | Ref | Ref | Ref | Ref |
| Student/Unpaid | 0.99 (0.79-1.25) | 0.74 (0.54-1.00) | 0.78 (0.52-1.13) | **^1^ 1.22 (1.00-1.47)** | 1.32 (0.87-1.94) |
| Not Working/Unemp. | 1.04 (0.87-1.23) | 0.92 (0.75-1.13) | 0.93 (0.72-1.18) | 1.07 (0.93-1.24) | 1.09 (0.79-1.48) |
| Retired | 0.98 (0.82-1.16) | 1.15 (0.95-1.38) | **^3^ 1.48 (1.21-1.82)** | 0.92 (0.80-1.06) | 0.76 (0.52-1.09) |
| **Children at home** |  |  |  |  |  |
| No | Ref | Ref | Ref | Ref | Ref |
| Yes | 1.09 (0.95-1.24) | 1.05 (0.90-1.22) | 0.93 (0.77-1.13) | 0.98 (0.88-1.10) | 0.80 (0.62-1.02) |
| **Education** |  |  |  |  |  |
| High School or below | Ref | Ref | Ref | Ref | Ref |
| Some Col. / Assoc. D. | 1.10 (0.90-1.34) | 0.87 (0.73-1.04) | 0.99 (0.76-1.29) | 0.97 (0.84-1.13) | **^3^ 3.13 (1.85-5.72)** |
| Bachelor’s D. + | **^1^ 1.25 (1.04-1.52)** | **^3^ 0.72 (0.61-0.87)** | 0.97 (0.76-1.26) | 1.05 (0.91-1.22) | **^3^ 3.68 (2.21-6.68)** |
| **Political affiliation** |  |  |  |  |  |
| Democrat | Ref | Ref | Ref | Ref | Ref |
| Republican | **^3^ 0.13 (0.11-0.16)** | **^3^ 33.56 (25.60-44.87)** | **^3^ 0.09 (0.06-0.12)** | **^3^ 0.70 (0.61-0.80)** | **^3^ 0.26 (0.17-0.39)** |
| Other | **^3^ 0.39 (0.34-0.46)** | **^3^ 5.94 (4.39-8.15)** | **^3^ 0.25 (0.20-0.32)** | **^2^ 1.23 (1.09-1.40)** | 1.09 (0.84-1.40) |
| Prefer not to say | **^3^ 0.36 (0.31-0.42)** | **^3^ 8.94 (6.68-12.17)** | **^3^ 0.23 (0.18-0.29)** | **^2^ 1.25 (1.10-1.42)** | 0.78 (0.57-1.04) |

**^1^** p<.05; **^2^** p<.01; **^3^** p<.001; ^Odds of using source compared to those not using source, adjusting for all other covariates in tables.
