# Supplementary material for: Trends and Predictors of COVID-19 Information Sources and Their Relationship With Knowledge and Beliefs Related to the Pandemic: Nationwide Cross-Sectional Study
Source: JMIR Public Health Surveill. 2020 Oct 8;6(4):e21071. doi: 10.2196/21071 (PMC7546863; doi:10.2196/21071)
Supplement: Multimedia Appendix 2 [file publichealth_v6i4e21071_app2.docx]

**Table 2: Changes in COVID-19 Information source between March and April, 2020, n = 11,242**

|  | *March (%)* | *April (%)* | *Percentage change* | *Adjusted^ Odds Ratio (Ref=March)* |
| --- | --- | --- | --- | --- |
| **Source group** |  |  |  |  |
| Traditional Media | 93.3 | 91.2 | -2.1 | **^3^ 0.68 (0.58-0.79)** |
| Government | 91.9 | 83.8 | -8.1 | **^3^ 0.41 (0.36-0.47)** |
| Online Media | 87.9 | 84.2 | -3.7 | **^3^ 0.75 (0.67-0.84)** |
| Interpersonal Sources | 73.5 | 68.2 | -5.3 | **^3^ 0.78 (0.71-0.85)** |
| Doctor | 55.4 | 46.1 | -9.3 | **^3^ 0.66 (0.61-0.72)** |
| Religious Leader | 9.7 | 5.5 | -4.2 | **^3^ 0.59 (0.50-0.69)** |
| **Most trusted source*** |  |  |  |  |
| Government | 53.3 | 36.9 | -16.4 | **^3^ 0.51 (0.47-0.56)** |
| TV | 3.9 | 5.6 | 1.7 | **^2^ 1.40 (1.15-1.70)** |
| Social Media | 1.1 | 1.3 | 0.2 | 1.25 (0.86-1.83) |
| Newspaper | 5.0 | 8.1 | 3.1 | **^3^ 1.50 (1.27-1.78)** |
| Websites | 3.3 | 6.9 | 3.6 | **^3^ 2.08 (1.72-2.53)** |
| Friends | 0.3 | 0.6 | 0.3 | 1.83 (1.00-3.49) |
| Doctor | 29.5 | 33.9 | 4.4 | **^3^ 1.25 (1.15-1.36)** |
| Radio | 0.6 | 1.6 | 1.0 | **^3^ 2.54 (1.68-3.93)** |
| Partner | 2.0 | 4.0 | 2.0 | **^3^ 2.21 (1.72-2.87)** |
| Family | 0.9 | 1.3 | 0.4 | **^1^ 1.71 (1.14-2.61)** |

**^1^** p<.05; **^2^** p<.01; **^3^** p<.001; *due to small sample size of those identifying religious leaders as their most trusted source (n=8), these were removed for analysis

^ Adjusted for sex, age, race, region, type of residence, working status, children, education, political affiliation
